# Characterization of *Epichloë coenophiala* within the US: are all tall fescue endophytes created equal?

**DOI:** 10.3389/fchem.2014.00095

**Published:** 2014-11-04

**Authors:** Carolyn A. Young, Nikki D. Charlton, Johanna E. Takach, Ginger A. Swoboda, Michael A. Trammell, David V. Huhman, Andrew A. Hopkins

**Affiliations:** The Samuel Roberts Noble Foundation, Forage Improvement DivisionArdmore, OK, USA

**Keywords:** *Neotyphodium coenophialum*, fescue toxicosis, ergot alkaloids, Kentucky-31 (KY31), endophyte diversity

## Abstract

Tall fescue (*Lolium arundinaceum*) is a valuable and broadly adapted forage grass that occupies approximately 14 million hectares across the United States. A native to Europe, tall fescue was likely introduced into the US around the late 1800's. Much of the success of tall fescue can be attributed to *Epichloë coenophiala* (formerly *Neotyphodium coenophialum*) a seed borne symbiont that aids in host persistence. *Epichloë* species are capable of producing a range of alkaloids (ergot alkaloids, indole-diterpenes, lolines, and peramine) that provide protection to the plant host from herbivory. Unfortunately, most tall fescue within the US, commonly referred to as “Kentucky-31” (KY31), harbors the endophyte *E. coenophiala* that causes toxicity to grazing livestock due to the production of ergot alkaloids. Molecular analyses of tall fescue endophytes have identified four independent associations, representing tall fescue with *E. coenophiala, Epichloë* sp. FaTG-2, *Epichloë* sp. FaTG-3, or *Epichloë* sp. FaTG-4. Each of these *Epichloë* species can be further distinguished based on genetic variation that equates to differences in the alkaloid gene loci. Tall fescue samples were evaluated using markers to simple sequence repeats (SSRs) and alkaloid biosynthesis genes to determine endophyte strain variation present within continental US. Samples represented seed and tillers from the Suiter farm (Menifee County, KY), which is considered the originating site of KY31, as well as plant samples collected from 14 states, breeder's seed and plant introduction lines (National Plant Germplasm System, NPGS). This study revealed two prominent *E. coenophiala* genotypes based on presence of alkaloid biosynthesis genes and SSR markers and provides insight into endophyte variation within continental US across historical and current tall fescue samples.

## Introduction

### History of KY31 tall fescue and discovery of endophyte

**Figure 1:**
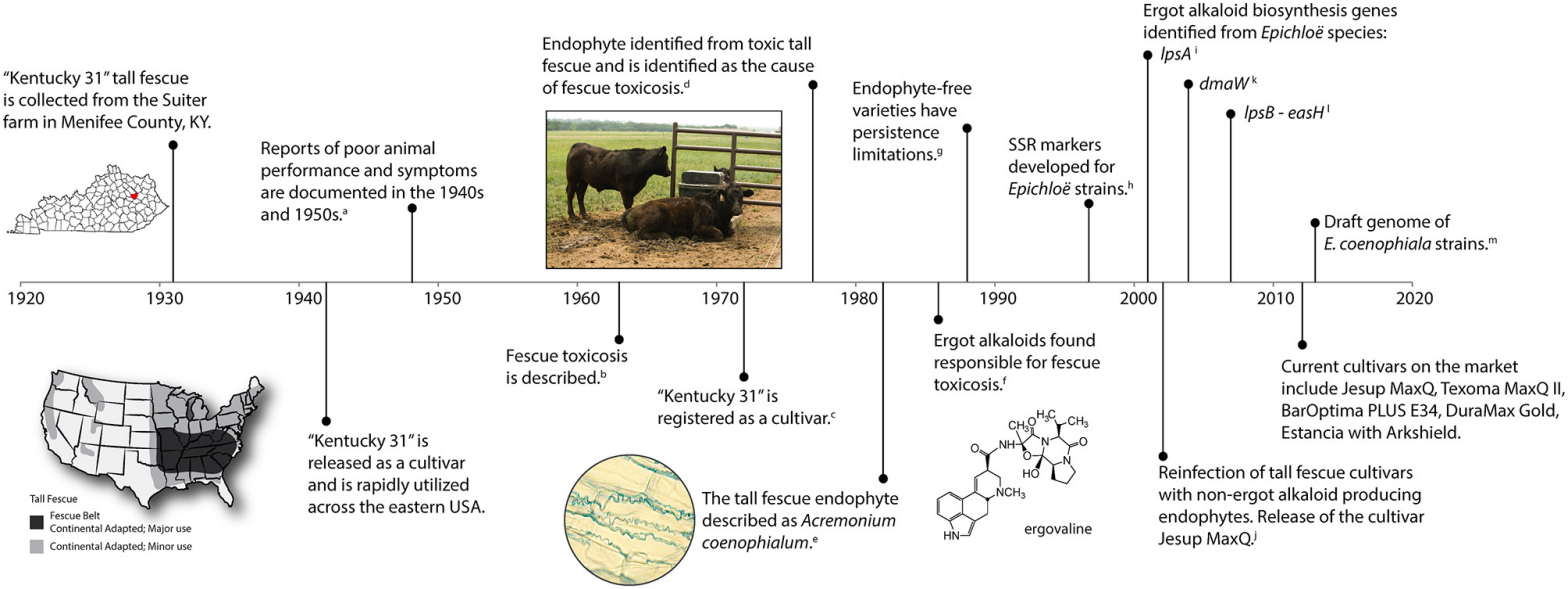
**A timeline of significant events associated with endophyte-infected tall fescue**. Citations include a, Cunningham, 1948; b, Jacobson et al., 1963; c, Fergus and Buckner, 1972; d, Bacon et al., 1977; e, Morgan-Jones and Gams, 1982; f, Lyons et al., 1986; g, Pedersen et al., 1990; h, Groppe et al., 1995; Moon et al., 1999; i, Panaccione et al., 2001; j, Bouton et al., 2002; k, Wang et al., 2004; l, Fleetwood et al., 2007; m, Schardl et al., 2013b.

Tall fescue [*Lolium arundinaceum* (Schreb.) Darbysh. syn *Festuca arundinaceae* Shreb.] was introduced into the United States from Europe in the 1800's and is considered an important cool season perennial forage crop (Hoveland, 2009). Tall fescue is widely adapted to the eastern United States spanning 14 million hectares (35 million acres) with the fescue belt considered the major region of adaptation and use (Figure 1) (Ball et al., 1993). A timeline representing significant research events of tall fescue is shown in Figure 1 and outlined below.

One of the most well known tall fescue cultivars, “Kentucky-31” (KY31), was collected in 1931 by Dr. E. N. Fergus (University of Kentucky) on a farm owned by William Suiter (Menifee County, KY) (Fergus and Buckner, 1972). KY31 gained wide acceptance as a grass with excellent agronomic attributes under difficult growth conditions, such as drought and poor soils. The KY31 ecotype was released in 1942, but was not officially registered as a cultivar until 1972 (Fergus and Buckner, 1972). Subsequently, the persistence and success of KY31 was attributed to the presence of the systemic fungal endophyte, *Epichloë coenophiala*. It was estimated that 90% of all tall fescue pastures in the US are endophyte infected (Siegel et al., 1985). The fitness benefits the endophyte provides include drought tolerance, improved competitive ability (Arachevaleta et al., 1989; West et al., 1993; Malinowski and Belesky, 2000), as well as protection from herbivores through the production of bioactive alkaloids (Clay et al., 1985; Bacon et al., 1986).

Unfortunately, although KY31 was known as a persistent cultivar, reports of poor animal performance when grazing this forage began in the 1940s (Cunningham, 1949; Jacobson et al., 1963). Animals that grazed on tall fescue suffered maladies such as fescue foot, fat necrosis, and fescue toxicosis (Bush et al., 1979). Cattle experiencing fescue toxicosis can exhibit rough hair coats, heat stress (wallowing in mud), elevated rectal temperatures, vasoconstriction, suppressed appetite, reduced prolactin levels, poor growth (lower average daily gains), and a reduction in calving rates (Hoveland et al., 1983; Hemken et al., 1984; Stuedemann and Hoveland, 1988; Roberts and Andrae, 2004; Caldwell et al., 2013). Symptoms of fescue foot resembled those observed from ergot alkaloid toxicity seen with *Claviceps purpurea* on rye suggesting that an ergot alkaloid might be responsible for toxicity (Yates, 1971). However, although the syndrome was first described in 1963 (Jacobson et al., 1963) it took another decade before an endophyte was suggested as the causal agent. In 1977, a fungal endophyte was identified in toxic tall fescue as the likely culprit of these symptoms (Bacon et al., 1977), which was later confirmed by the production of ergot alkaloids by the fungus (Porter et al., 1979; Lyons et al., 1986; Bacon, 1988). The endophyte grows systemically through the upper plant parts and is maternally inherited in the seed (Siegel et al., 1984; Schardl, 2001).

### Tall fescue endophyte description

Initially, Bacon et al. (1977) identified the tall fescue endophytes as *E. typhina*, which was later renamed *Acremonium coenophialum* to acknowledge the anomorphic state of *Epichloë* species (Morgan-Jones and Gams, 1982). Christensen and Latch (1991) described variation among isolates of *A. coenophialum* from tall fescue, and in 1993 the taxonomy of these endophytes was described (Christensen et al., 1993). The genus *Acremonium* was reclassified using phylogenetic analyses and *A. coenophialum* was renamed *Neotyphodium coenophialum* (Glenn et al., 1996). Finally, under the nomenclatural rule changes for fungi *Neotyphodium* is now included in the genus *Epichloë* resulting in the change to *E. coenophiala* (Leuchtmann et al., 2014). *E. coenophiala* is considered an asexual hybrid, phylogenetically described as a triparental hybrid with inferred ancestral progenitors from *E. festucae, E. typhina* subsp. *poae*, and the *Lolium*-associated endophyte (LAE) (Tsai et al., 1994; Moon et al., 2004).

*E. coenophiala*, like many *Epichloë* species, is capable of producing a variety of bioactive secondary metabolites. The four described classes of alkaloids produced by *Epichloë* species are ergot alkaloids, indole-diterpenes, lolines, and peramine (Siegel et al., 1990). Ergot alkaloids (e.g., ergovaline) and the indole-diterpene, lolitrem B, have been shown to have anti-mammalian activity causing fescue toxicosis (Bacon et al., 1977) and ryegrass staggers (Fletcher and Harvey, 1981), respectively. Peramine is considered an insect feeding deterrent (Johnson et al., 1985; Rowan and Latch, 1994) and the lolines have been documented for their potent insecticidal activity (Bush et al., 1997). *E. coenophiala* as a species complex can produce all four classes of alkaloids (Table 1). However, the most commonly produced alkaloids are peramine, lolines, and ergovaline (Christensen et al., 1993; Leuchtmann et al., 2000; Schardl et al., 2013b).

**Table 1:** ***Epichloë* species and genotype variation associated with endophytes of tall fescue**.

**Endophyte species**	**Endophyte genotype^a^**	**Ploidy**	**Progenitors^b^**	**Minimum marker set to distinguish species genotypes**					**Predicted chemotype class^d^**
				**Mating type**	**Ergot alkaloid *EAS***	**Indole-diterpene *IDT*/*LTM***	**Loline *LOL***	**Peramine *PER*^c^**	
*E. coenophiala*	Profile 1	3x	Efe (II) × LAE (Vb) × Ety (Ib)	AAA	*dmaW, lpsB*		*lolC, lolA*	*perA*-A2	*EAS, LOL, PER*
*E. coenophiala*	Profile 2	3x	Efe (II) × LAE (Vb) × Ety (Ib)	AAA	*dmaW, lpsB*	*idtQ*	*lolC, lolA*	*perA*-A2	*EAS, LOL, PER*
*E. coenophiala*	Profile 3	3x	Efe (II) × LAE (Vb) × Ety (Ib)	AAA	*dmaW, lpsB*	*idtG, idtQ*	*lolC, lolA*	*perA*-A2	*EAS, IDT, LOL, PER*
*E. coenophiala*	Profile 4	3x	Efe (II) × LAE (Vb) × Ety (Ib)	AAA		*idtG, idtQ*	*lolC, lolA*	*perA*-A2	*IDT, LOL, PER*
*Epichloë* sp. FaTG-2	Profile 1	2x	Efe (II) × LAE (Vb)	BB	*dmaW, lpsB*	*idtG, idtQ, ltmJ*		*perA*-A2, Δ*perA*-A2	*EAS, LTM, PER*
*Epichloë* sp. FaTG-2	Profile 2	2x	Efe (II) × LAE (Vb)	AB	*dmaW, lpsB*	*idtG, idtQ*		perA-A2, ΔperA-A2	*EAS, IDT, PER*
*Epichloë* sp. FaTG-2	Profile 3	2x	Efe (II) × LAE (Vb)	AB	*dmaW, lpsB*	*idtG, idtQ, ltmJ*		*perA*-A2, Δ*perA*-A2	*EAS, LTM, PER*
*Epichloë* sp. FaTG-3	Profile 1	2x	LAE (Vb) × Ety (Ia)	AA		*idtG, idtQ*	*lolC, lolA*	perA-A2, ΔperA-A2	*IDT, LOL, PER*
*Epichloë* sp. FaTG-3	Profile 2	2x	LAE (Vb) × Ety (Ia)	AA			*lolC, lolA*	perA-A2, ΔperA-A2	*LOL, PER*
*Epichloë* sp. FaTG-4	Profile 1	2x	LAE (Vb) × Ety (Ia)	AB	*dmaW, lpsB*	*idtG*		perA-A2	*EAS, PER*
*Epichloë* sp. FaTG-4	Profile 2	2x	LAE (Vb) × Ety (Ia)	AB	*dmaW, lpsB*	*idtG, idtQ*		perA-A2	*EAS, IDT, PER*

a Based on designations from Takach and Young (2014) and draft genome sequences of FaTG-2 isolates NFe45079 and NFe45115.

b Efe (II), E. festucae (mating population II); LAE, Lolium associated endophyte (mating population Vb); Ety (Ia), E. typhina (mating population 1a); Ety (Ib), E. typhina (mating population 1b); Mating population as designated from Leuchtmann et al. (2014).

c The perA-A2 marker is designed to the second adenylation domain. Some isolates have a deletion in this domain as represented by ΔperA-A2 (Takach et al., 2012).

d Predicted chemotype class represents the class of genes that are found in the genome and do not always represent a functionally active locus. EAS, ergot alkaloids; IDT, indole diterpenes; LTM, lolitrem B; LOL, lolines; PER, peramine.

Considerable research has been conducted to understand the biosynthesis of these bioactive compounds including identification and characterization of the gene products required for the biosynthesis of each alkaloid class (Panaccione et al., 2001; Wang et al., 2004; Spiering et al., 2005, 2008; Tanaka et al., 2005; Young et al., 2006, 2009; Fleetwood et al., 2007; Saikia et al., 2012; Pan et al., 2014). This has been supported with genome sequences, including draft genome sequences of three *E. coenophiala* strains (Schardl et al., 2013a,b). This research has provided an understanding of why *Epichloë* species can have diverse alkaloid profiles and provided the sequence to develop markers for mating type and key alkaloid biosynthesis genes to genetically evaluate endophyte diversity *in planta* (Charlton et al., 2012, 2014; Takach et al., 2012; Takach and Young, 2014).

To date, tall fescue is known to form associations with four taxonomic groups, *E. coenophiala, Epichloë* sp. FaTG-2, *Epichloë* sp. FaTG-3, and *Epichloë* sp. FaTG-4 that vary based on ploidy (either 2x or 3x) and progenitors (Table 1). Initially tall fescue endophytes were distinguished by morphology and isozyme analysis to establish taxonomic groupings, and variation was also seen with the production of peramine, ergovaline, lolitrem B, and lolines (Christensen et al., 1993). Phylogenetic analyses were able to define the relationships of each taxonomic group to distinguish the ancestral progenitors of these hybrid species (Schardl et al., 1991, 2013b; Moon et al., 2004). The most studied of these species is *E. coenophiala*, the endophyte first identified in KY31.

Isozyme analyses of *E. coenophiala* isolates from within the US indicated that very little variation existed within this species (Leuchtmann and Clay, 1990). Although isozyme analysis can reflect endophyte diversity, this analysis requires pure cultures and thus is limited by the number of samples per tall fescue line that can be screened. Genetic analysis can now be performed directly with endophyte infected plant material using high throughput systems (Takach and Young, 2014). Markers have recently been used to determine the genetic diversity between tall fescue endophyte isolates and also evaluate their potential for alkaloid production (Ekanayake et al., 2012; Takach et al., 2012; Takach and Young, 2014). In fact, variation of mating type and alkaloid genes determined by PCR could be enough to allow placement of tall fescue endophytes into distinct genotype groups associated with each *Epichloë* species (Table 1) (Takach and Young, 2014). At least four unique *E. coenophiala* genotypes are easily distinguishable among tall fescue sourced originally from Europe and the Mediterranean basin (Ekanayake et al., 2012; Takach and Young, 2014).

Literature surrounding endophyte-infected tall fescue that causes fescue toxicosis often refers to *E. coenophiala* as the common toxic endophyte. The objective of this study was to compare the endophytes within tall fescue cultivars, varieties and ecotypes from the US using markers to SSRs and alkaloid biosynthesis genes to identify and characterize these endophytes. We have determined endophyte diversity across historical and current tall fescue samples to evaluate the endophyte diversity that may exist across the US.

## Materials and methods

### Biological materials

Tall fescue plant material was provided by researchers in Alabama, Arkansas, Georgia, Kentucky, Missouri, Mississippi, New York, Ohio, Oklahoma, Pennsylvania, South Carolina, Tennessee, Texas, and West Virginia (Table 2). Plants were maintained in a space plant nursery under rain-fed conditions or in the greenhouse at the Samuel Roberts Noble Foundation, Ardmore, Oklahoma. Each researcher was requested to provide at least 10 independent plants from fields known to cause fescue toxicosis or thought to contain the common toxic endophyte. Tall fescue seed stocks (PI lines) were sourced from the National Plant Germplasm System (NPGS). Georgia-5 (GA-5) seed was provided by JH Bouton and other seed stocks were sourced from the Samuel Roberts Noble Foundation tall fescue (NFTF) breeding program and designated NFTF.

**Table 2:** **Sources of tall fescue plants from US collection**.

**State**	**County**	**Plant designation^a^**	**No. of plants maintained**	**No. plants that died**	**No. of endophyte-infected plants**	**Endophyte genotypes present**
AL	Dallas	Black belt station	21	0	21	2-1, 2-2
AR	Nevada	Prescott	6	0	6	1-1, 2-1
AR	Hempstead	Deanne	6	0	6	2-1
FL, GA, MD, NY		GA-5	10	2	7	1-1, 1-2, 2-1, 2-3
GA	Walker	Walker county	12	0	8	2-1
GA	Wayne	Jesup	9	2	6	2-1
KY	Mennifee	Suiter farm	10	1	9	1-1, 2-1, 2-4
KY	Caldwell	Pennyrile	10	1	4	2-1
MO	Camden	Lake Farm	12	0	9	2-1
MO	Camden	Ford place	8	0	8	2-1
MO	Camden	Tiny's place	4	0	3	2-1
MO	St. Louis	Hencken	6	0	6	2-1
MS	Oktibbeha	Starksville	9	0	9	2-1, 2-4
NY	Allegany	Alfred	5	0	5	2-1
OH	Coshocton	NAEW graze	6	0	6	1-1, 2-1
OH	Coshocton	NAEW hay	6	0	5	2-1
OK	Carter	NFTF 1000	9	0	7	2-1
OK	Woodward	NFTF 1100	9	1	7	2-1
OK	Hughes	Calvin	2	0	1	2-1
PA	Huntingdon	Soder	1	0	1	1-?
PA	Huntingdon	Soder (Petersburg)	3	0	3	3-1
PA	Centre	Everhart	1	0	1	2-3
PA	Centre	JRE state college	5	1	4	2-1, 2-2
SC	Anderson	NFTF 1491	10	0	5	2-1
TN	Henderson	Lexington	4	0	4	2-1
TN	Henderson	Natchez trace	7	0	6	1-1, 2-1
TX	Fannin	Fannin	4	0	4	2-1
TX	Fannin	NFTF 1492	10	3	6	2-1, 2-2
TX	Crosby, Lubbock, Lamb, Briscoe	NFTF 1230	9	0	8	1-1, 2-1
TX	Kerr	NFTF 1480	9	1	8	2-1
WV	Raleigh	Roscoe upper	10	0	8	1-1, 2-1
WV	Raleigh	Roscoe middle	10	1	7	2-1
WV	Raleigh	Roscoe lower	10	3	6	2-1, 2-4
WV	Raleigh	Reba	10	3	5	2-1

a Plant designation refers to landmark or site location or plant breeding line information (NFTF).

### DNA isolation and endophyte genotyping

Total DNA from individual seeds or tillers from stock plants were isolated using QIAGEN MagAttract 96 DNA Plant Core Kit (Qiagen Inc., Valencia, CA). Primers specific for *tefA*, tef1-exon1d (5′-GGGTAAGGACGAAAAGACTCA-3′) and tef1-exon5u-1 (5′-CGGCAGCGATAATCAGGATAG-3′) (Craven et al., 2001; Moon et al., 2002) were used to detect the presence of endophyte. A minimum set of key alkaloid genes and two mating type genes were chosen to differentiate the *E. coenophiala* endophytes present in continental tall fescue based on the previous study by Takach and Young (2014). The markers were designed to *mtAC* and *mtBA* mating type genes, *dmaW* and *lpsB* for representatives of the *EAS* locus, *lolC* and *lolA* for representatives of the *LOL* locus, *idtG* and *idtQ* for representatives of the *IDT* locus, and *perA* second adenylation domain (*perA*-A2) for *PER*. Multiplex PCR was performed in a total volume of 25 μL containing 3 μL DNA, 1.0 U GoTaq™ DNA Polymerase (Promega Corp., Madison, WI), 1× Green GoTaq™ Reaction Buffer containing 1.5 mM MgCl_2_, 0.2 mM of each dNTP (Promega Corp.), and 1 μM of each primer as described previously (Takach et al., 2012; Charlton et al., 2014; Takach and Young, 2014). The cycling parameters were an initial denaturation step for 1 min at 94 C, 30 cycles of denaturation at 94 C for 15 s, annealing at 56 C for 30 s, extension at 72 C for 45 s, followed by a final synthesis step at 72 C for 10 min.

PCR of the microsatellite B10 and B11 loci (Moon et al., 1999) were used to differentiate endophytes within an *E. coenophiala* profile. For SSR analysis, one primer at each locus was end labeled with a fluorescent phosphoramidite dye. Specifically, primers B10.1 was labeled with 2′-chloro-7′phenyl-1,4-dichloro-6-carboxy-fluorescein (VIC) and B11.1 was labeled with 2′-chloro-5′-fluoro-7′,8′-benzo-1,4-dichloro-6-carbo xyfluorescein (NED) (Life Technologies, Carlsbad, CA). PCR was performed in a total volume of 10 μL containing diluted DNA (approximately 0.5 ng), 0.75 U Platinum *Taq* DNA Polymerase (Life Technologies), 1× PCR Buffer (-Mg), 1.5 mM MgCl_2_, 100 nM of each dNTP (Promega Corp.) and 200 nM of each primer. The cycling parameters were an initial denaturation step for 4 min at 94 C, 35 cycles of denaturation at 94 C for 30 s, annealing at 60 C for 30 s, extension at 72 C for 30 s, followed by a final synthesis step at 72 C for 7 min. PCR products (1.5 μL of a 1:10 dilution) were added to 9.9 μL of Hi-Di formamide and 0.1 μL of GeneScan™ 500 LIZ™ size standard (Life Technologies). Samples were denatured at 94 C for 5 min prior to separation on an ABI 3730 DNA Analyzer. Data analysis was performed using Peak Scanner Software v1.0 (Applied Biosystems).

### Ergovaline analysis

Pseudostems were collected from greenhouse grown plants, lyophilized and ground into a fine powder and stored at −20°C. Ergovaline concentrations were measured in duplicate using 10 ± 0.10 mg of tissue for each endophyte infected sample. Samples were extracted in 200 μL of methanol containing 0.005 mg/mL dihydroergotamine tartrate salt (Sigma-Aldrich, St. Louis, MO) for 3 h and then centrifuged at 1800 × *g* for 5 min. Each sample was analyzed for the presence of ergovaline using an ACQUITY ultra-performance liquid chromatography (UPLC) system (Waters Corporation, Milford, MA) as described previously (Takach et al., 2012). Seed extracts in which the ergovaline content was previously quantified (A. M. Craig, Endophyte Testing Laboratory, Oregon State University) were used as standards for quantification. Concentrations used to generate the standard curve included 0, 50, 97, 500, 1000, and 2000 ppb.

The linear standard curve was plotted as ergovaline:ergotamine peak area ratio vs. the actual amount of ergovaline. Standards and samples were analyzed in duplicate and their values averaged.

## Results and discussion

### Evaluation of historical tall fescue endophytes from the united states

The cultivar KY31 is well known for causing fescue toxicosis and has been distributed over much of the eastern United States (Figure 1). KY31 was established from an ecotype collection from the Suiter farm in Menifee County, KY (released as a cultivar in 1943) and source material (PI 531431) from this location was deposited into NPGS in 1991. Other cultivars, developed after KY31, have also contributed to the dissemination of endophyte-infected tall fescue (Pedersen and Sleper, 1988). In particular, Alta (cultivar in 1945) was considered a successful cultivar in northeast US and was likely interbred with KY31 (Asay et al., 1979). Seed from other early tall fescue cultivars such as Alta, Kenmont (cultivar in 1963), Kenwell (cultivar in 1965), Kenhy (cultivar in 1977), and Missouri 96 (cultivar in 1977) (Pedersen and Sleper, 1988) were also included in our study (see Table 3 for NPGS deposition dates). Studies that have included some of these early cultivars have subsequently indicated they were infected with a common toxic endophyte (Bacon et al., 1977; Cornell et al., 1982; Siegel et al., 1984; Pedersen and Sleper, 1988). We also evaluated more recent cultivars and germplasm from the NFTF breeding program that contain common toxic endophyte (Table 3).

**Table 3:** **Characterization of endophytes from Kentucky 31 tall fescue seed in US based on microsatellite variation**.

**Seed stock**	**Year^a^**	**Seeds tested**	**%E+ (number)**	**%E– (number)**	**%E+ Ecoe profile 1^b^ (number)**	**%E+ Ecoe profile 2^b^ (number)**
PI 561431 - KY31	1991	46	91% (42)	9% (4)	48% (22)^c^	43% (20)^d^
KY31 commercial seed^e^	2011	48	79% (38)	21% (10)	17% (8)^c^	63% (30)^d^
KY31 - SW Missouri	2008	46	98% (45)	2% (1)	0% (0)	98% (45)^d^
PI 596701 - Missouri 96	1979	24	0	100 (24)	0% (0)	0% (0)
PI 578714 - Kenmont	1963	24	0	100 (24)	0% (0)	0% (0)
PI 574521 - Kenwell	1965	22	0	100 (22)	0% (0)	0% (0)
PI 434051 - Kenhy	1979	24	0	100 (24)	0% (0)	0% (0)
PI 601020 - Johnstone^f^	1983	24	0	100 (24)	0% (0)	0% (0)
PI 578712 - Alta	1962	48	0	100 (48)	0% (0)	0% (0)
Jesup E+	2003	10	100% (10)	0% (0)	0% (0)	100% (10)
NFTF 1000 - PDF E+^g^	1998	36	100% (36)	0% (0)	8% (3)	92% (33)
NFTF 1011 - PDF E+^g^	2007	12	100% (12)	0% (0)	33% (4)	67% (8)
NFTF 1041 - PDF E+^g^	2010	12	100% (12)	0% (0)	0% (0)	100% (12)

a The year the seed was purchased, generated or when it entered NPGS.

b As determined by markers consistent with E. coenophiala profile 1 and 2 from Table 1.

c SSR B10 = 161, 170, 184; B11 = 147, 191.

d SSR B10 = 152, 161, 178; B11 = 171, 195.

e The KY31 commercial seed Tri-Star Seed Co., Inc. located in Spring Hill, KS was purchased from the Tractor Supply Company, Ardmore, Oklahoma, in July 2011.

f Johnstone was released as an endophyte free cultivar (Buckner et al., 1983).

g NFTF 1011 and NFTF 1041 represent selections from the original NFTF 1000 (also known as PDF E+ Hopkins et al., 2011) from Oklahoma.

Seed from each cultivar or line were analyzed for endophyte infection and genetic variation (Table 3). Unfortunately many of the seed samples sourced from NPGS were endophyte-free or had levels less than 5%. Since endophyte viability can be compromised during storage (Siegel et al., 1985; Rolston and Agee, 2007) there was no guarantee these seeds would represent the endophyte status of the original plant material. Only the three KY31 samples, Jesup E+ and NFTF breeding lines were positive for endophyte presence. In addition, two endophyte genotypes, *E. coenophiala* profile 1 and profile 2 that vary based on presence of *IDT* genes, could be distinguished in four of these seed samples. However, the percentage of each endophyte strain varied in each seed lot. Four of the tested seed lines, KY31 (from Missouri), Jesup E+ and NFTF 1041 may represent an *E. coenophiala* profile 2 monoculture, or contain a low incidence of *E. coenophiala* profile 1 as the number of seeds tested for some lines were low (Table 3).

Lines that were selected from NFTF 1000-PDF (NFTF 1011 selected from PDF for vigorous growth, high forage yield and digestibility, and NFTF 1041 selected from PDF for high digestibility by marker assisted selection) showed different ratios of each *E. coenophiala* profile (Table 3). This may show the influence an endophyte strain, which is maintained in the maternal line, can have on selectable traits such as persistence and vigor if the endophyte provides a host advantage. As such, it would be interesting to evaluate the host genetic shifts under selection, with and without endophytes, while also following selection of different endophyte strains.

### Endophyte analysis from development of the cultivar Georgia-5

**Figure 2:**
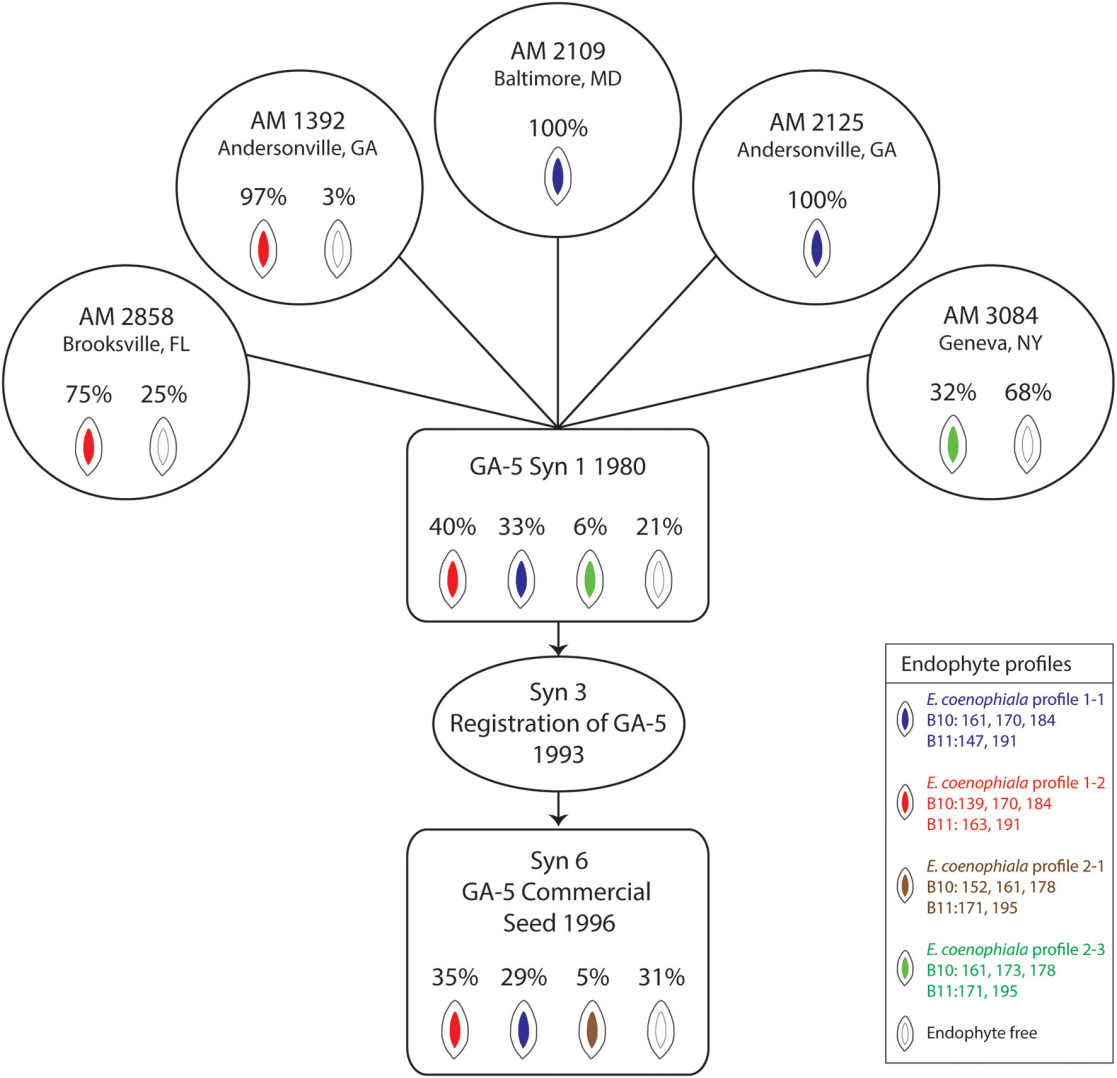
***Epichloë coenophiala* profiles and infection frequencies of seed used in the development of the “Georgia 5” tall fescue cultivar**. The five clones consisted of AM 1392, AM 2109, AM 2125, AM 2858, and AM 3084. The synthetic 3 generation represents the breeders seed increase from the five clones (Bouton et al., 1993b).

Analysis of endophyte variation within a population provides an opportunity to evaluate material incorporated through a tall fescue breeding pipeline and eventually released for commercial production (Figure 2). The GA-5 cultivar was developed as a synthetic endophyte-infected cultivar with superior forage yield and persistence in the Southern Coastal Plains that had potential to replace KY31 (Bouton et al., 1993b). The cultivar was established from five clones and was shown to be 75% endophyte infected (Bouton et al., 1993b). We evaluated seed from the original five clones (each clone having originated from a different location) using markers to SSRs and alkaloid biosynthesis genes to determine the initial infection rates of each clone and identify which *E. coenophiala* profiles were present. The endophyte status of the originating lines varied from 32 to 100% infection, and the endophyte profiles were consistent within the seed sample from each clone. Three independent endophyte genotypes (based on SSRs) were identified within the clones (Figure 2). Seed from synthetic 1 established in 1980 was also tested for endophyte infection and identification, and all three endophyte genotypes were represented within this sample with an overall endophyte infection level of 79% (Figure 2). In 1993, GA-5 was registered as a cultivar (Bouton et al., 1993b) and subsequently released commercially in 1996. When we evaluated a seed stock from the commercial line the overall infection level was 69% and two of the three expected endophyte SSR profiles were identified within the sample. However, an additional endophyte genotype (B10 = 152, 161, 178 and B11 = 171, 195) was present in 5% of the seed sample (Figure 2) that has likely arisen from contamination later in production. The level of endophyte free seed increased from Syn 1 (21%) to Syn 6 (31%) and may indicate that production favored this part of the population. Unfortunately we were unable to detect the endophyte genotype profile 2 with B10 = 161, 173, 178 and B11 = 171, 195, which may be due to the number of seeds that were tested.

### Endophyte diversity from US tall fescue collections

To further examine tall fescue endophyte diversity within the US, collections of tall fescue plants from 14 eastern states were evaluated for endophyte presence. In total, 246 tall fescue plants were screened using markers for SSRs B10 and B11, and the minimum set of alkaloid biosynthesis genes to distinguish the different *E. coenophiala* genetic profiles. Of the 246 plants initially screened, 204 (83%) were endophyte-infected. All of the endophyte-infected samples could be amplified with primer sets to *mtAC, perA*, and the *LOL* and *EAS* markers, and samples only varied with the presence of *IDT* markers. Three *E. coenophiala* genetic profiles were identified (*E. coenophiala* profiles 1, 2, and 3; Table 4).

**Table 4:** **Characterization of endophytes from tall fescue plants in US based on alkaloid profiles and microsatellite variation**.

**Endophyte genotype**	**B10 allele sizes (bp)**	**B11 allele sizes (bp)**	***PER*^a^**	***EAS*^a^**		***LOL*^a^**		***IDT*^a^**		**No. of plants**	**States**
			***perA*-A2**	***dmaW***	***lpsB***	***lolC***	***lolA***	***idtG***	***ltmQ***		
*E. coenophiala* profile 1-1	161, 170, 184	147, 191	+	+	+	+	+			11	AR, KY, OH, TN, TX, WV
*E. coenophiala* profile 1-2	139, 170, 184	163, 191	+	+	+	+	+			2	GA
*E. coenophiala* profile 2-1	152, 161, 178	171, 195	+	+	+	+	+		+	172	AL, AR, GA, KY, MO, MS, NY, OH, OK, PA, SC, TN, TX, WV
*E. coenophiala* profile 2-2	152, 161, 178	183, 195	+	+	+	+	+		+	3	AL, TX
*E. coenophiala* profile 2-3	161, 173, 178	171, 195	+	+	+	+	+		+	3	GA (NY)^b^, PA
*E. coenophiala* profile 2-4	161, 173, 178	171, 210	+	+	+	+	+		+	4	KY, MS, WV
*E. coenophiala* profile 3-1	161, 170, 178	155, 163	+	+	+	+	+	+	+	3	PA

a The + indicates that a PCR fragment was detected with primers designed to the gene.

b Identified out of GA-5 and represents the clone from NY (see Figure 2).

Further analysis using the B10 and B11 SSR markers showed additional variation within the genetic profiles. There were two unique SSR profiles for genotype 1, four SSR profiles for genotype 2 and one SSR profile for genotype 3. *E. coenophiala* profile 1 is consistent with the same pattern of the KY31 endophyte *E. coenophiala* strain e19 (Schardl et al., 1991; Takach and Young, 2014), while *E. coenophiala* profile 2 is more highly represented in the samples.

The two most common SSR marker patterns were also identified in the KY31 seed (Table 3). Interestingly, of the nine plants that were collected from the original Suiter farm (Table 2), one plant showed a third unique SSR profile in common with endophytes found in West Virginia and Mississippi. Only one set of plants from Pennsylvania produced *E. coenophiala* genetic profile 3, but unfortunately knowledge about the tall fescue planted at this site was unavailable. This Pennsylvania endophyte-infected tall fescue is the only set of plants that were likely to produce indole-diterpenes.

Plants with unique *E. coenophiala* profiles were selected from the nursery and maintained in the greenhouse. Ergovaline was analyzed from pseudostems of 25 plants representing the seven unique *E. coenophiala* profiles. Each of the plant-endophyte associations produced ergovaline (Table 5), indicating that the endophyte in these lines would all be considered a common toxic endophyte. The ergovaline levels generated under greenhouse conditions were also extremely high, well above the threshold of 400–750 ppb required for toxicity in livestock (Hovermale and Craig, 2001). Although the levels tested from these plants are high, it may simply reflect they were well maintained plants subjected to regular fertilization, which is known to result in higher levels of ergovaline production (Rottinghaus et al., 1991).

**Table 5:** **Ergovaline concentrations of tall fescue infected with different *Epichloë coenophiala* genotypes**.

**Endophyte genotype^a^**	**B10 allele sizes (bp)**	**B11 allele sizes (bp)**	**No. of plants tested**	**States represented**	**Range of [Ergovaline] (ppm)**
*E. coenophiala* profile 1-1	161, 170, 184	147, 191	5	GA, KY, TN, TX, WV	2.2–7.1
*E. coenophiala* profile 1-2	139, 170, 184	163, 191	1	GA	4.1
*E. coenophiala* profile 2-1	152, 161, 178	171, 195	11	AL, GA, KY, MO, MS, PA, TN, TX, WV	1.7–7.8
*E. coenophiala* profile 2-2	152, 161, 178	183, 195	2	AL, TX	1.2–9.3
*E. coenophiala* profile 2-3	161, 173, 178	171, 195	3	GA (NY)^b^, PA	2.4–5.5
*E. coenophiala* profile 2-4	161, 173, 178	171, 210	2	KY, MS	1.9–8.4
*E. coenophiala* profile 3-1	161, 170, 178	155, 163	1	PA	4.2

a Based on designations from Takach and Young (2014).

b Identified from the GA-5 line and represents the clone from NY (see Figure 2).

This study provides a snapshot of common toxic endophyte-infected tall fescue across the eastern US. The endophyte genotypes we identified were consistent with samples from Europe where tall fescue was originally sourced (Takach and Young, 2014). Endophyte genotype diversity was present at the origin of KY31 but it appears *E. coenophiala* genotype 2 is the dominant endophyte genotype across the US. This endophyte was also prevalent in other breeding populations such as Jesup E+ and NFTF 1000. Given the sample numbers we have selected from each location, it is still possible that other endophyte genotypes are present at low frequencies. Although KY31 is believed to be the predominant source of tall fescue in the US, other sources were developed and planted at various times and locations, and it is possible that during this process one endophyte genotype provided a selectable advantage. Indeed, if we look at NFTF 1011, a selection from the NFTF 1000 line (Table 3), we see a shift in endophyte genotype that may be due to specific selection pressures.

### New cultivars with selected endophytes

To overcome fescue toxicosis, researchers initially removed the endophyte from toxic tall fescue. Unfortunately, studies that evaluated the endophyte role on tall fescue performance found endophyte-infected lines had better persistence and greater yield than endophyte-free lines (Pedersen et al., 1990; Bouton et al., 1993a, 2002; West et al., 1993; Gunter and Beck, 2004). As expected, when production of ergot alkaloids was low or not present, livestock also had better overall performance and increased average daily gains (Stuedemann and Hoveland, 1988; Gunter and Beck, 2004). An ideal solution to capture both endophyte associated plant persistence and reduced livestock toxicity was to identify an endophyte strain that retained traits for plant persistence, but did not produce the alkaloids toxic to livestock. Analysis of tall fescue across its natural distribution of Europe and the Mediterranean basin has shown diversity of both the endophyte (Christensen et al., 1993; Ekanayake et al., 2012; Takach and Young, 2014) and its plant host (Hand et al., 2012); subsequently this diversity has been exploited to establish selected endophyte-infected tall fescue with low mammalian toxicity (reviewed in Bouton, 2009; Johnson et al., 2013; Young et al., 2013). Typically these endophytes lack many or all of the genes at the *EAS* locus required for ergot alkaloid biosynthesis (e.g., *E. coenophiala* profile 4, Table 1) (Takach and Young, 2014), although some endophytes have been selected for lower ergot alkaloid production.

Initial success with selected endophytes was observed when the endophyte strain AR542 (known commercially as MaxQ and MaxP in the US and Australia, respectively) was inoculated into Jesup and GA-5 (Bouton et al., 2002). Agronomic evaluations indicated selected endophyte-tall fescue associations provided the benefits of endophyte infection (stand persistence) with animal performance similar to endophyte-free tall fescue (Bouton et al., 2002; Parish et al., 2003; Gunter and Beck, 2004). Jesup MaxQ (Pennington Seed, Inc.) was the first commercial tall fescue cultivar to be released and used by farmers containing a selected endophyte. Additional endophyte-infected tall fescue lines have since been established and evaluated for both plant and animal performance (Roberts and Andrae, 2004; Hopkins et al., 2010; Parish et al., 2013; Beck et al., 2014). One of the most recently released cultivars, Texoma MaxQ II, is the result of breeding for plant persistence with an ecotypic selection that was subsequently inoculated with the endophyte strain AR584 (MaxQ II) that does not cause livestock toxicity (Hopkins et al., 2010, 2011). Current commercially available cultivars of selected endophyte-tall fescue associations include: Jesup MaxQ (Pennington Seed, Inc.), Texoma MaxQ (Pennington Seed Inc.), BarOptima Plus E34 (Barenbrug), Duramax Gold (DLF International Seeds), and Estancia with ArkShield (MountainView Seeds).

## Conclusion

Endophytes have clearly affected the success of tall fescue within the US, from the prevalence of the common toxic endophyte through to advancing cultivars with selected endophytes. Many farmers have learned to manage the effects of fescue toxicosis through pasture management techniques, but now there are also options for eliminating toxicity by pasture replacement. The availability of elite tall fescue lines infected with selected endophytes allows farmers to provide nutritious, non-toxic feed for their livestock without fear of toxic repercussions. Climate change will likely increase the dependency of tall fescue monocultures to rely upon endophytes to provide drought tolerance and protection from insect pests. As more selected endophyte-infected cultivars enter the marketplace to replace tall fescue pastures containing common toxic endophytes, we will be able to examine the constancy of these symbiotic associations over time. Utilization of molecular markers will provide effective methods to identify endophyte strains within tall fescue cultivars, varieties and ecotypes, and help distinguish endophyte friend from foe.

### Conflict of interest statement

The authors declare that the research was conducted in the absence of any commercial or financial relationships that could be construed as a potential conflict of interest.

## References

[B1] Arachevaleta M. Bacon C. W. Hoveland C. S. Radcliffe D. E. (1989). Effect of the tall fescue endophyte on plant response to environmental stress. Agron. J. 81, 83–90 10.2134/agronj1989.00021962008100010015x

[B2] Asay K. Frakes R. V. Buckner R. C. (1979). Breeding and cultivars, in Tall Fescue, eds Buckner R. Bush L. (Madison, WI: American Society of Agronomy, Crop Science Society of America, Soil Science Society of America), 111–139.

[B3] Bacon C. Porter J. Robbins J. Luttrell E. (1977). *Epichloë typhina* from toxic tall fescue grasses. Appl. Environ. Microbiol. 34, 576–581. 931377 10.1128/aem.34.5.576-581.1977 PMC242703

[B4] Bacon C. W. (1988). Procedure for isolating the endophyte from tall fescue and screening isolates for ergot alkaloids. Appl. Environ. Microbiol. 54, 2615–2618. 3214150 10.1128/aem.54.11.2615-2618.1988 PMC204344

[B5] Bacon C. W. Lyons P. C. Porter J. K. Robbins J. D. (1986). Ergot toxicity from endophyte-infected grasses: a review. Agron. J. 78, 106–116. 10.2134/agronj1986.00021962007800010023x7608021

[B6] Ball D. M. Pederson J. Lacefield G. D. (1993). The tall-fescue endophyte. Am. Sci. 81, 370–379.

[B7] Beck P. Stewart C. Gray H. Gadberry M. Gunter S. Young C. (2014). Using tall fescue in a complementary grazing program for Spring calving beef cows in Southern Arkansas. Prof. Anim. Sci. 30, 423–431.

[B8] Bouton J. (2009). Deployment of novel endophytes in the tall fescue commercial seed trade, in Tall Fescue for the Twenty-First Century, Agronomy Monograph 53, eds Fribourg H. Hannaway D. West C. (Madison, WI: American Society of Agronomy, Crop Science Society of America, Soil Science Society of America), 367–375.

[B9] Bouton J. Gates R. Belesky D. Owsley M. (1993a). Yield and persistence of tall fescue in the southeastern coastal plain after removal of its endophyte. Agron. J. 85, 52–55 10.2134/agronj1993.00021962008500010011x

[B10] Bouton J. Gates R. Hill G. Owsley M. Wood D. (1993b). Registration of ‘Georgia 5’ tall fescue. Crop Sci. 33, 1405 10.2135/cropsci1993.0011183X003300060059x

[B11] Bouton J. H. Latch G. C. Hill N. S. Hoveland C. S. McCann M. A. Watson R. H.. (2002). Reinfection of tall fescue cultivars with non-ergot alkaloid–producing endophytes. Agron. J. 94, 567–574. 10.2134/agronj2002.056715827262

[B12] Buckner R. Boling J. Burrus P. Bush L. Hemken R. (1983). Registration of Johnstone tall fescue (Reg. No. 23). Crop Sci. 23, 399–400 10.2135/cropsci1983.0011183X002300020057x

[B13] Bush L. Boling J. Yates S. (1979). Animal disorders, in Tall Fescue, eds Buckner R. C. Bush L. P. (Madison, WI: American Society of Agronomy, Crop Science Society of America, Soil Science Society of America), 247–292.

[B14] Bush L. P. Wilkinson H. H. Schardl C. L. (1997). Bioprotective alkaloids of grass-fungal endophyte symbioses. Plant Physiol. 114, 1–7. 12223685 10.1104/pp.114.1.1 PMC158272

[B15] Caldwell J. D. Coffey K. P. Jennings J. A. Philipp D. Young A. N. Tucker J. D. Hubbell D. S. 3rd.. (2013). Performance by spring and fall-calving cows grazing with full, limited, or no access to toxic *Neotyphodium coenophialum*-infected tall fescue. J. Anim. Sci. 91, 465–476. 10.2527/jas.2011-4603 22785163

[B16] Charlton N. D. Craven K. D. Afkhami M. E. Hall B. H. Ghimire S. R. Young C. A. (2014). Interspecific hybridization and bioactive alkaloid variation increases diversity in endophytic *Epichloë* species of *Bromus laevipes*. FEMS Microbiol. Ecol. 90, 276–289. 10.1111/1574-6941.12393 25065688

[B17] Charlton N. D. Craven K. D. Mittal S. Hopkins A. A. Young C. A. (2012). *Epichloë canadensis*, a new interspecific epichloid hybrid symbiotic with Canada wildrye (*Elymus canadensis*). Mycologia 104, 1187–1199. 10.3852/11-403 22675049

[B18] Christensen M. Latch G. (1991). Variation among isolates of *Acremonium* endophytes (*A. coenophialum* and possibly *A. typhinum)* from tall fescue *(Festuca arundinacea)*. Mycol. Res. 95, 1123–1126 10.1016/S0953-7562(09)80558-3

[B19] Christensen M. Leuchtmann A. Rowan D. Tapper B. (1993). Taxonomy of *Acremonium* endophytes of tall fescue (*Festuca arundinacea*), meadow fescue (*F. pratensis)* and perennial ryegrass *(Lolium perenne)*. Mycol. Res. 97, 1083–1092. 10.1016/S0953-7562(09)80509-111693372

[B20] Clay K. Hardy T. N. Hammond A. M. (1985). Fungal endophytes of grasses and their effects on an insect herbivore. Oecologia 66, 1–5 10.1007/BF00378545 28310805

[B21] Cornell C. Garner G. Yates S. Bell S. (1982). Comparative fescue foot potential of fescue varieties. J. Anim. Sci. 55, 180–184.

[B22] Craven K. D. Hsiau P. T. W. Leuchtmann A. Hollin W. Schardl C. L. (2001). Multigene phylogeny of *Epichloë* species, fungal symbionts of grasses. Ann. Mo. Bot. Gard. 88, 14–34 10.2307/2666129

[B23] Cunningham I. (1948). Tall fescue grass is poison for cattle. N.Z. J. Agric. 77, 519.

[B24] Cunningham I. (1949). A note on the cause of tall fescue lameness in cattle. Aust. Vet. J. 25, 27–28 10.1111/j.1751-0813.1949.tb04752.x

[B25] Ekanayake P. N. Hand M. L. Spangenberg G. C. Forster J. W. Guthridge K. M. (2012). Genetic diversity and host specificity of fungal endophyte taxa in fescue pasture grasses. Crop Sci. 52, 2243–2252 10.2135/cropsci2011.12.0664

[B26] Fergus E. Buckner R. C. (1972). Registration of Kentucky 31 Tall Fescue (Reg. No. 7). Crop Sci. 12, 714 10.2135/cropsci1972.0011183X001200050061x

[B27] Fleetwood D. J. Scott B. Lane G. A. Tanaka A. Johnson R. D. (2007). A complex ergovaline gene cluster in *Epichloë* endophytes of grasses. Appl. Environ. Microbiol. 73, 2571–2579. 10.1128/AEM.00257-07 17308187 PMC1855613

[B28] Fletcher L. R. Harvey I. C. (1981). An association of a *Lolium* endophyte with ryegrass staggers. N.Z. Vet. J. 29, 185–186. 10.1080/00480169.1981.34839 6950332

[B29] Glenn A. E. Bacon C. W. Price R. Hanlin R. T. (1996). Molecular phylogeny of *Acremonium* and its taxonomic implications. Mycologia 88, 369–383 10.2307/3760878

[B30] Groppe K. Sanders I. Wiemken A. Boller T. (1995). A microsatellite marker for studying the ecology and diversity of fungal endophytes (*Epichloë* spp.) in grasses. Appl. Environ. Microbiol. 61, 3943–3949. 8526508 10.1128/aem.61.11.3943-3949.1995 PMC167701

[B31] Gunter S. Beck P. (2004). Novel endophyte-infected tall fescue for growing beef cattle. J. Anim. Sci. 82, E75–E82. 15471817 10.2527/2004.8213_supplE75x

[B32] Hand M. L. Cogan N. O. Forster J. W. (2012). Molecular characterisation and interpretation of genetic diversity within globally distributed germplasm collections of tall fescue (*Festuca arundinacea* Schreb.) and meadow fescue (*F. pratensis Huds.)*. Theor. Appl. Genet. 124, 1127–1137. 10.1007/s00122-011-1774-6 22222441

[B33] Hemken R. Jackson J. Jr. Boling J. (1984). Toxic factors in tall fescue. J. Anim. Sci. 58, 1011–1016. 6373703 10.2527/jas1984.5841011x

[B34] Hopkins A. Young C. Butler T. Bouton J. (2011). Registration of ‘Texoma’MaxQ II tall fescue. J. Plant Regist. 5, 14–18 10.3198/jpr2010.02.0082crc

[B35] Hopkins A. Young C. Panaccione D. Simpson W. Mittal S. Bouton J. (2010). Agronomic performance and lamb health among several tall fescue novel endophyte combinations in the south-central USA. Crop Sci. 50, 1552–1561 10.2135/cropsci2009.08.0473

[B36] Hoveland C. Schmidt S. King C. Odom J. Clark E. McGuire J. (1983). Steer performance and association of *Acremonium coenophialum* fungal endophyte on tall fescue pasture. Agron. J. 75, 821–824 10.2134/agronj1983.00021962007500050021x

[B37] Hoveland C. S. (2009). Origin and history, in Tall Fescue for the 21st Century, eds Fribourg H. Hannaway D. West C. (Madison, WI: Amer Society of Agronomy), 3–10.

[B38] Hovermale J. T. Craig A. M. (2001). Correlation of ergovaline and lolitrem B levels in endophyte-infected perennial ryegrass (*Lolium perenne*). J. Vet. Diagn. Invest. 13, 323–327. 10.1177/104063870101300407 11478604

[B39] Jacobson D. Miller W. Seath D. Yates S. Tookey H. Wolff I. (1963). Nature of fescue toxicity and progress toward identification of the toxic entity. J. Dairy Sci. 46, 416–422 10.3168/jds.S0022-0302(63)89066-9

[B40] Johnson L. J. de Bonth A. C. Briggs L. R. Caradus J. R. Finch S. C. Fleetwood D. J. (2013). The exploitation of epichloae endophytes for agricultural benefit. Fungal Divers. 60, 171–188 10.1007/s13225-013-0239-4

[B41] Johnson M. C. Dahlman D. L. Siegel M. R. Bush L. P. Latch G. C. Potter D. A.. (1985). Insect feeding deterrents in endophyte-infected tall fescue. Appl. Environ. Microbiol. 49, 568–571. 16346751 10.1128/aem.49.3.568-571.1985 PMC373550

[B42] Leuchtmann A. Bacon C. W. Schardl C. L. White J. F. Tadych M. (2014). Nomenclatural realignment of *Neotyphodium* species with genus *Epichloë*. Mycologia 106, 202–215. 10.3852/106.2.202 24459125

[B43] Leuchtmann A. Clay K. (1990). Isozyme variation in the *Acremonium/Epichloë* fungal endophyte complex. Phytopathology 80, 1133–1139 10.1094/Phyto-80-1133

[B44] Leuchtmann A. Schmidt D. Bush L. (2000). Different levels of protective alkaloids in grasses with stroma-forming and seed-transmitted *Epichloë*/*Neotyphodium* endophytes. J. Chem. Ecol. 26, 1025–1036 10.1023/A:1005489032025

[B45] Lyons P. C. Plattner R. D. Bacon C. W. (1986). Occurrence of peptide and clavine ergot alkaloids in tall fescue grass. Science 232, 487–489. 10.1126/science.3008328 3008328

[B46] Malinowski D. P. Belesky D. P. (2000). Adaptations of endophyte-infected cool-season grasses to environmental stresses: mechanisms of drought and mineral stress tolerance. Crop Sci. 40, 923–940 10.2135/cropsci2000.404923x

[B47] Moon C. D. Craven K. D. Leuchtmann A. Clement S. L. Schardl C. L. (2004). Prevalence of interspecific hybrids amongst asexual fungal endophytes of grasses. Mol. Ecol. 13, 1455–1467. 10.1111/j.1365-294X.2004.02138.x 15140090

[B48] Moon C. D. Miles C. O. Jarlfors U. Schardl C. L. (2002). The evolutionary origins of three new *Neotyphodium* endophyte species from grasses indigenous to the Southern Hemisphere. Mycologia 94, 694–711. 10.2307/3761720 21156542

[B49] Moon C. D. Tapper B. A. Scott B. (1999). Identification of *Epichloë* endophytes in planta by a microsatellite-based PCR fingerprinting assay with automated analysis. Appl. Environ. Microbiol. 65, 1268–1279. 10049893 10.1128/aem.65.3.1268-1279.1999 PMC91174

[B50] Morgan-Jones G. Gams W. (1982). Notes on Hyphomycetes. XLI. An endophyte of *Festuca arundinacea* and the anamorph of *Epichloe typhina*, new taxa in one of two new sections of *Acremonium*. Mycotaxon 15, 311–318.

[B51] Pan J. Bhardwaj M. Faulkner J. R. Nagabhyru P. Charlton N. D. Higashi R. M.. (2014). Ether bridge formation in loline alkaloid biosynthesis. Phytochemistry 98, 60–68. 10.1016/j.phytochem.2013.11.015 24374065 PMC3929955

[B52] Panaccione D. G. Johnson R. D. Wang J. Young C. A. Damrongkool P. Scott B.. (2001). Elimination of ergovaline from a grass–*Neotyphodium* endophyte symbiosis by genetic modification of the endophyte. Proc. Natl. Acad. Sci. U.S.A. 98, 12820–12825. 10.1073/pnas.221198698 11592979 PMC60137

[B53] Parish J. A. McCann M. A. Watson R. H. Paiva N. N. Hoveland C. S. Parks A. H.. (2003). Use of nonergot alkaloid-producing endophytes for alleviating tall fescue toxicosis in stocker cattle. J. Anim. Sci. 81, 2856–2868. 14601890 10.2527/2003.81112856x

[B54] Parish J. A. Parish J. R. Best T. F. Boland H. T. Young C. A. (2013). Effects of selected endophyte and tall fescue cultivar combinations on steer grazing performance, indicators of fescue toxicosis, feedlot performance, and carcass traits. J. Anim. Sci. 91, 342–355. 10.2527/jas.2011-4725 23048138

[B55] Pedersen J. Lacefield G. Ball D. (1990). A review of the agronomic characteristics of endophyte-free and endophyte-infected tall fescue. Appl. Agric. Res. 5, 188–194.

[B56] Pedersen J. Sleper D. (1988). Considerations in breeding endophyte-free tall fescue forage cultivars. J. Prod. Agric. 1, 127–132 10.2134/jpa1988.0127

[B57] Porter J. K. Bacon C. W. Robbins J. D. (1979). Ergosine, ergosinine, and chanoclavine I from *Epichloë typhina.* J. Agric. Food Chem. 27, 595–598. 10.1021/jf60223a045 447932

[B58] Roberts C. Andrae J. (2004). Tall fescue toxicosis and management. Crop Manage. 3 10.1094/CM-2004-0427-01-MG

[B59] Rolston M. Agee C. (2007). Delivering quality seed to specification-the USA and NZ novel endophyte experience, in Proceedings of the 6th International Symposium on Fungal Endophytes of Grasses. Christchurch, New Zealand. Grassland Research and Practice Series, eds Popay A. Thom E. (Dunedin, NZ: NZ Grassl. Assoc. Inc.), 229–231.

[B60] Rottinghaus G. E. Garner G. B. Cornell C. N. Ellis J. L. (1991). HPLC method for quantitating ergovaline in endophyte-infested tall fescue: seasonal variation of ergovaline levels in stems with leaf sheaths, leaf blades, and seed heads. J. Agric. Food Chem. 39, 112–115 10.1021/jf00001a022

[B61] Rowan D. Latch G. (1994). Utilization of endophyte-infected perennial ryegrasses for increased insect resistance, in Biotechnology of Endophytic Fungi of Grasses, eds Bacon C. W. White J. F. J. (Boca Raton, FL: CRC Press), 169–183.

[B62] Saikia S. Takemoto D. Tapper B. A. Lane G. A. Fraser K. Scott B. (2012). Functional analysis of an indole-diterpene gene cluster for lolitrem B biosynthesis in the grass endosymbiont *Epichloë festucae.* FEBS Lett. 586, 2563–2569. 10.1016/j.febslet.2012.06.035 22750140

[B63] Schardl C. L. (2001). *Epichloë festucae* and related mutualistic symbionts of grasses. Fungal Genet. Biol. 33, 69–82. 10.1006/fgbi.2001.1275 11456460

[B64] Schardl C. L. Liu J. White J. F. Jr. Finkel R. A. An Z. Siegel M. R. (1991). Molecular phylogenetic relationships of nonpathogenic grass mycosymbionts and clavicipitaceous plant pathogens. Plant Syst. Evol. 178, 27–41 10.1007/BF00937980

[B65] Schardl C. L. Young C. A. Hesse U. Amyotte S. G. Andreeva K. Calie P. J.. (2013a). Plant-symbiotic fungi as chemical engineers: multi-genome analysis of the Clavicipitaceae reveals dynamics of alkaloid loci. PLoS Genet. 9:e1003323. 10.1371/journal.pgen.1003323 23468653 PMC3585121

[B66] Schardl C. L. Young C. A. Pan J. Florea S. Takach J. E. Panaccione D. G.. (2013b). Currencies of mutualisms: sources of alkaloid genes in vertically transmitted Epichloae. Toxins 5, 1064–1088. 10.3390/toxins5061064 23744053 PMC3717770

[B67] Siegel M. Latch G. Bush L. Fannin F. Rowan D. Tapper B.. (1990). Fungal endophyte-infected grasses: alkaloid accumulation and aphid response. J. Chem. Ecol. 16, 3301–3315. 10.1007/BF00982100 24263431

[B68] Siegel M. R. Johnson M. C. Varney D. Nesmith W. Buckner R. Bush L. P. (1984). A fungal endophyte in tall fescue: incidence and dissemination. Phytopathology 74, 932–937 10.1094/Phyto-74-932

[B69] Siegel M. R. Latch G. C. M. Johnson M. C. (1985). *Acremonium* fungal endophytes of tall fescue and perennial ryegrass: significance and control. Plant Dis. 69, 179–181.

[B70] Spiering M. J. Faulkner J. R. Zhang D. X. Machado C. Grossman R. B. Schardl C. L. (2008). Role of the LolP cytochrome P450 monooxygenase in loline alkaloid biosynthesis. Fungal Genet. Biol. 45, 1307–1314. 10.1016/j.fgb.2008.07.001 18655839

[B71] Spiering M. J. Moon C. D. Wilkinson H. H. Schardl C. L. (2005). Gene clusters for insecticidal loline alkaloids in the grass-endophytic fungus *Neotyphodium uncinatum*. Genetics 169, 1403–1414. 10.1534/genetics.104.035972 15654104 PMC1449547

[B72] Stuedemann J. A. Hoveland C. S. (1988). Fescue endophyte: history and impact on animal agriculture. J. Prod. Agric. 1, 39–44 10.2134/jpa1988.0039

[B73] Takach J. E. Mittal S. Swoboda G. A. Bright S. K. Trammell M. A. Hopkins A. A.. (2012). Genotypic and chemotypic diversity of *Neotyphodium* endophytes in tall fescue from Greece. Appl. Environ. Microbiol. 78, 5501–5510. 10.1128/AEM.01084-12 22660705 PMC3406137

[B74] Takach J. E. Young C. A. (2014). Alkaloid genotype diversity of tall fescue endophytes. Crop Sci. 54, 667–678. 10.2135/cropsci2013.06.042322660705

[B75] Tanaka A. Tapper B. A. Popay A. Parker E. J. Scott B. (2005). A symbiosis expressed non-ribosomal peptide synthetase from a mutualistic fungal endophyte of perennial ryegrass confers protection to the symbiotum from insect herbivory. Mol. Microbiol. 57, 1036–1050. 10.1111/j.1365-2958.2005.04747.x 16091042

[B76] Tsai H. F. Liu J. S. Staben C. Christensen M. J. Latch G. C. Siegel M. R.. (1994). Evolutionary diversification of fungal endophytes of tall fescue grass by hybridization with *Epichloë* species. Proc. Natl. Acad. Sci. U.S.A. 91, 2542–2546. 10.1073/pnas.91.7.2542 8172623 PMC43405

[B77] Wang J. Machado C. Panaccione D. G. Tsai H. F. Schardl C. L. (2004). The determinant step in ergot alkaloid biosynthesis by an endophyte of perennial ryegrass. Fungal Genet. Biol. 41, 189–198. 10.1016/j.fgb.2003.10.002 14732265

[B78] West C. Izekor E. Turner K. Elmi A. (1993). Endophyte effects on growth and persistence of tall fescue along a water-supply gradient. Agron. J. 85, 264–270 10.2134/agronj1993.00021962008500020019x

[B79] Yates S. (1971). Toxin-producing fungi from fescue pasture. Microb. Toxins 7, 191–206 10.1016/B978-0-12-046507-1.50012-4

[B80] Young C. A. Felitti S. Shields K. Spangenberg G. Johnson R. D. Bryan G. T.. (2006). A complex gene cluster for indole-diterpene biosynthesis in the grass endophyte *Neotyphodium lolii.* Fungal Genet. Biol. 43, 679–693. 10.1016/j.fgb.2006.04.004 16765617

[B81] Young C. A. Hume D. E. McCulley R. L. (2013). Forages and pastures symposium: fungal endophytes of tall fescue and perennial ryegrass: pasture friend or foe? J. Anim. Sci. 91, 2379–2394. 10.2527/jas.2012-5951 23307839

[B82] Young C. A. Tapper B. A. May K. Moon C. D. Schardl C. L. Scott B. (2009). Indole-diterpene biosynthetic capability of *Epichloë* endophytes as predicted by *ltm* gene analysis. Appl. Environ. Microbiol. 75, 2200–2211. 10.1128/AEM.00953-08 19181837 PMC2663189

